# Identification and local manipulation of bone marrow vasculature during intravital imaging

**DOI:** 10.1038/s41598-020-63533-3

**Published:** 2020-04-14

**Authors:** Takayuki Morikawa, Shinpei Tamaki, Shinya Fujita, Makoto Suematsu, Keiyo Takubo

**Affiliations:** 1 Department of Stem Cell Biology, Research Institute, National Center for Global Health and Medicine, Tokyo, 162-8655 Japan; 2 Department of Biochemistry, Keio University School of Medicine, Tokyo, 160-8582 Japan

**Keywords:** Imaging the immune system, Haematopoietic stem cells, Stem-cell niche

## Abstract

Physiological regulation of blood flow in bone marrow is important to maintain oxygen and glucose supplies but also the physiological hypoxic state of the hematopoietic stem cell (HSC) niche. However, regulatory mechanisms underlying microcirculation in the bone marrow (BM) niche remain unclear. Here, we identify vessels functioning in control of blood flow in bone marrow and assess their contractility. To evaluate contractile potential of Alexa Fluor 633 (AF633; an arterial marker)-positive vessels, we performed immunohistochemistry for α-smooth muscle actin (α-SMA) and found it expressed around AF633^+^ vessels in the femoral and calvarial marrow. To validate AF633^+^ vessel contractility, we developed a simple system to locally administer vasoactive agents that penetrate BM through transcalvarial vessels. After exposure of the calvarial surface to FITC-dextran (70 kDa), FITC intensity in calvarial bone marrow gradually increased. When we evaluated the effect of transcalvarial administration (TCA) of norepinephrine (NE) on vascular tone of AF633^+^ arteries and behavior of transplanted blood cells, NE administration decreased artery diameter and transendothelial migration of transplanted cells, suggesting that adrenergic signaling regulates the HSC niche microcirculation and blood cell migration into the BM via effects on BMarteries. We conclude that TCA is a useful tool for bone marrow research.

## Introduction

Hematopoietic stem cells (HSCs) self-renew and differentiate to produce various types of blood cells during an animal’s lifetime. In adult mammals, HSCs mainly reside in bone marrow (BM) and are maintained by an adjacent microenvironment called the BM niche, consisting of niche cells such as endothelial cells, CXCL12-abundant reticular cells or mesenchymal stem cells and their progenies, non-myelinating Schwann cells, and megakaryocytes^1–8^. Overall, niche cells provide cytokines, chemokines, adhesion molecules, and extracellular matrix proteins that preserve HSC function and maintain HSC number in BM. Moreover, the mobilization of HSC is promoted by sympathetic signaling via osteoblast and osteocyte-dependent mechanism^9,10^.

Much of our current understanding of homeostasis in many organs has emerged following use of multi-photon microscopy to visualize tissue-resident cells *in vivo*, a methodology that originated in the field of physiology^11,12^. Others have extended this technology to analysis of the murine calvarium to assess migratory behavior of osteoclasts and spatiotemporal intercellular interactionsofliving osteoblasts and osteoclasts^13–15^. Calvarial bioimaging has resulted in important advances in hematopoiesis research, in part by defining positional relationships between HSCs and niche cells^16,17^. For example, combining fluorescent angiographic techniques with analysis in mouse reporter lines in which hematopoietic stem/progenitor cells (HSPCs) express a fluorescent protein has enabled us to trace hemodynamics and HSPC movement through vascular walls in the BM^18,19^. Moreover, analysis of transgenic mice in which regulatory T cells (T_reg_s) express fluorescent protein demonstrated the immunological privilege of HSPCs^20^, and *in vivo* imaging revealed the dynamics of leukemic cells and how the BM environment is remodeled during leukemogenesis^21–23^. Overall, current *in vivo* imaging techniques are useful to study location, interaction, and transmigration of BM cells.

In addition to cellular niche components and related proteins, non-cellular factors in BM ,such as physiological hypoxia, function as a component of the niche^24,25^. Studies combining *in vivo* imaging with direct measurement of local concentration of oxygen (pO_2_) in the calvarial BM suggested that the pO_2_ inside blood vessels sharply drops after vessels enter the BM, an observation attributable to active O_2_ consumption by BM cells^26^. Nonetheless, although relatively hypoxic, BM blood vessels supply sufficient O_2_ levels to fulfill oxygen demands of both hematopoietic cells (HSCs) and niche cells. Thus, in addition to their angiocrine function in providing niche factors to HSCs^2,4^, BM blood vessels also function to maintain physiological pO_2_ in the HSC niche by modulating BM perfusion. How BM perfusion contributes to BM hematopoiesis is a critical question that has been difficult to answer for two reasons: (1) lack of a method to classify BM vessel subtypes *in vivo* without transgenes and (2) lack of methods to manipulate the calvarial BM during intravital imaging. In the case of the femoral BM, arterial blood is mainly supplied through trans-cortical vessels and the nutrient artery, and the BM vasculature is subdivided into arteries, arterioles and sinusoids^27^. In calvarial BM bioimaging, the vasculature is defined and classified by vessel diameter, velocity of red blood cells and red blood cell density^19^. Development of additional imaging techniques to identify and manipulate BM vessels would allow direct investigation of the effects of BM perfusion on hematopoiesis.

In this study, we refine conventional *in vivo* imaging techniques to manipulate the BM microenvironment during imaging. First, we used intravital staining to visualize vessel subtypes of calvarial BM and identified contractile arteries associated with sympathetic nerves. We then pharmacologically manipulated vessels by transcalvarial drug administration (TCA). TCA of norepinephrine (NE) contracted BM arteries and reduced blood flow without altering systemic circulation, indicating that BM blood flow is regulated by signals from sympathetic neurons. We conclude that TCA allows detailed manipulation of the microcirculation during intravital imaging.

## Results

### Visualization of contractile vessels during intravital imaging of mouse calvarial bone marrow

**Figure 1:**
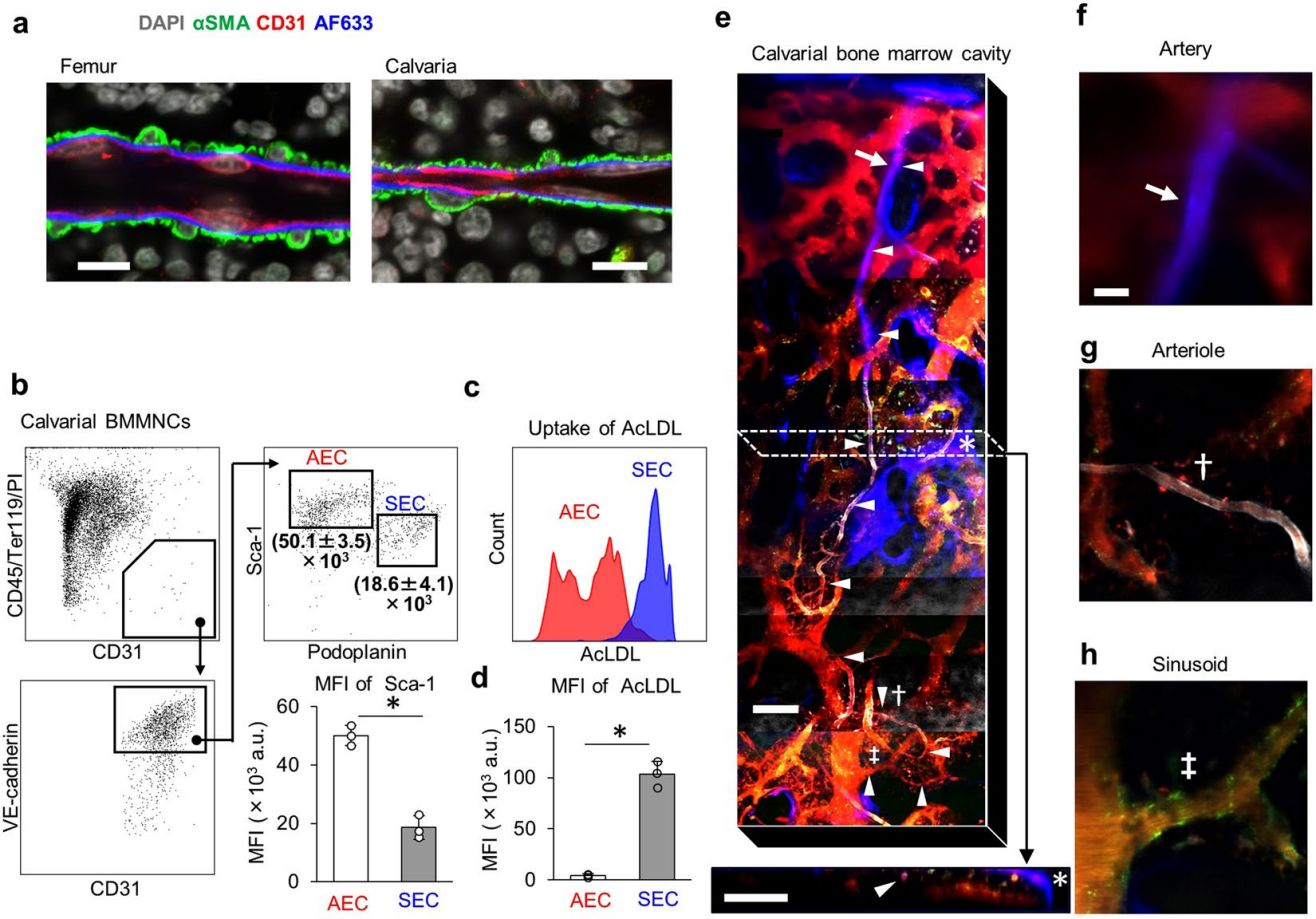
Classification of blood vessels in calvarial BM by *in vivo* intravital imaging. (**a**) *In vivo* staining of α-SMA^+^ vessels in BM by AF633. Shown is immunohistochemical localization of vascular smooth muscle cells and ECs in BM sections from femur and calvaria of an AF633-injected mouse. Scale bars = 10 μm. (**b**) Representative flow cytometry gating strategies used to identify AECs and SECs in calvarial BMMNCs. Calvarial BMMNCs were obtained 60 min after i.v. injection of anti-Sca-1 antibodies. Numbers under the gates of AEC and SEC indicate MFI of Sca-1 (mean ± SD, n = 3). Histogram of AECs and SECs stained with PE-Cy7-conjugated anti-Sca-1 antibody (mean ± SD, n = 3, *p < 0.05). (**c**,**d**) Histogram and MFI of AF488-conjugated AcLDL incorporated into AECs and SECs (mean ± SD, n = 3, *p < 0.05). (**e**) Shown is the calvarial BM cavity stained with AF633 (blue, arrow), BV450-conjugated anti-Sca-1 antibody (gray), and AF488-conjugated AcLDL (green) for *in vivo* intravital imaging. Arrowheads in upper and lower panels indicate a blood vessel that continues from an artery to a sinusoid. Lower panel shows an image acquired perpendicular to the plane of the upper panel and corresponding to the area inside dashed box shown in the upper panel. Asterisk indicates tissue stained by AF633 but considered a bone surface morphologically. Arrow, dagger and double-dagger indicate artery, arteriole and sinusoid shown in (**f–h**), respectively. Bar = 100 μm. (**f–h**) Arrow, dagger and double dagger indicate artery, arteriole and sinusoidal vessel, respectively. They also shown in (**e**). Bar = 20 μm.

To identify vessels that contribute to focal regulation of blood flow in the BM vascular network, we first searched for arterial markers useful for intravital imaging of mouse calvarial BM. The fluorophoreAlexa Fluor 633 hydrazide (AF633) reportedly binds to neocortical arteries and arterioles by specifically binding to elastin fibers^28^, and arteriolar vessels in calvarial BM are stained by intravenous injection of AF633^29^. A hallmark of contractile vessels is ensheathment of endothelial cells (ECs) by vascular smooth muscle cells (VSMCs). To determine whether AF633^+^ vessels were surrounded by VSMCs, we stained sections obtained from femoral and calvarial BM of AF633-injected mice with antibodies against α-smooth muscle actin (α-SMA) and CD31. We observed AF633^+^ elastin fibers in the basement membrane, the layer between VSMCs and ECs, in both femoral and calvarial BM (Fig. 1a). While perivascular cells (PVCs), which express the pericyte marker neural/glial antigen 2 (NG2, or CSPG4), may function in vessel contraction, NG2^+^ cells also resided on AF633^+^ vessels in calvarial BM (Fig. S1a,b)^30^. As previously reported by others^31^, von Willebrand factor was expressed in these AF633^+^ arteries (Fig S1c). These data suggest that AF633^+^ vessels have a contractile function driven by VSMCs and/or PVCs.

VSMC^+^ arteries connect to vascular networks consisting of arterioles and sinusoids. Femoral arterial ECs (AECs) and sinusoidal ECs (SECs) in BM are reportedly distinguished by cell surface expression of Sca-1 and podoplanin (PDPN) (Fig. S1d)^32^. These staining patterns allowed us to differentiate Sca-1^bright^ PDPN^−^ AECs from Sca-1^dim^ PDPN^+^ SECs in calvarial BM by FACS analysis (Fig. 1b). While some Sca-1^+^ vessels showed a α-SMA^low^ phenotype in calvarial BM, most Sca-1^bright^ vessels were associated with NG2^+^ PVCs (Fig. S1b,e). Overall, we conclude that, in addition to AF633^+^ arteries, calvarial Sca-1^bright^ vessels are associated with VSMCs and/or PVCs and potentially contractile and that Sca-1 antigen can serve as a marker of arterioles in intravital imaging of calvarial BM.

The sinusoidal vessel wall in mouse long bones actively incorporates acetylated-low density lipoprotein (AcLDL), as previously described (Fig. S1f)^33^. To characterize sinusoidal vessels of calvarial BM, we asked whether calvarial SECs were labeled by Alexa Fluor 488-conjugated (AF488) AcLDL* in vivo*. Flow cytometric analysis showed that mean fluorescence intensity (MFI) of AF488 of calvarial BM SECs was significantly higher than that of AECs (Fig. 1c,d). Consistent with previous reports, expression of mRNAs encoding stem cell factor and Ephrin B2 in AECs fractionated using AcLDL was higher than in SECs; on the other hand, VCAM-1 and E-selectin expression was lower in AECs than in SECs (Fig. S1g)^32^. Based on the fluorescence spectrum of individual dyes, we used AF633, Brilliant Violet 450 (BV450)-conjugated anti-Sca-1 antibody and AF488 AcLDL to identify calvarial BM arteries, arterioles and sinusoids for *in vivo* imaging studies (summarized in Table 1). For example, in Fig. 1e–h, arteries entering calvarial BM from the sagittal suture were stained strongly with AF633 (Figs. 1e,f and S1H). Since we also observed AF633 accumulation at the bone surface, we visualized tubular (arteries) and flat (bone surfaces) structures in calvarial BM using intravital imaging (Figs. 1e and S1i). AF633 staining of these vessels decreased as the distance from the sagittal suture, while Sca-1 expression remained high (Figs. 1e,g and S1b,e,j). As these arterioles went through several branching points, cell surface Sca-1 expression gradually decreased. Sca-1^dim^ vessels showed strong incorporation of AcLDL in their walls and flowed into large diameter-sinusoids (Figs. 1e,h and S1k, Table 1). Sca-1 and AcLDL were colocalized in the transitional zone (~40 μm) between arteriole and sinusoid. (Fig. S1k).

**Table 1:** Characteristics of each vessel type classified in this study.

	Vessel types classified in this study		
	Artery	Arteriole	Sinusoid
**Vascular markers**			
CD31 (IHC, FCM)	+	+	+
VE-cadherin (FCM)	+	+	+
AF633 (IVI, IHC)	+	−	−
α-SMA (IHC)	+	−	−
NG2 (IHC)	+	+	Low/−
Sca-1 (IVI, IHC, FCM)	+	+	Low
AcLDL (IVI, FCM)	−	−	+
Podoplanin (FCM)	−	−	+
**Vessel typesthat are considered identical in previous reports**			
Bixel *et al*., Cell Reports, 2017 (Skull bone)	Arterial vessel	Arterial vessel	Post-arterial / Intermediate / Sinusoidal capillaries
Itkin *et al*., Nature, 2016 (Long bone)	Artery	Endosteal arterioles	Sinusoid
Acar *et al*., Nature, 2015 (Long bone)	Arteriole	TZ vessel	Sinusoid
Kusumbe *et al*., Nature, 2015 (Long bone)	Artery	Arteriole	Type H/L capillary
**Dimensions and hemodynamic parameters**			
Diameter (μm)	10.9 ± 4.6 (6.9–17.2)	6.3 ± 1.2 (4.7–8.6)	20.8 ± 5.2 (14.0–28.1)
Velocity (mm/s)	2.0 ± 0.9 (1.1–3.1)	2.1 ± 1.4 (1.5–5.5)	0.2 ± 0.1 (0.1–0.3)
RBC flux (pL/s)	59.6 ± 82.7 (12.8–183.4)	14.9 ± 13.5 (3.5–44.9)	17.9 ± 11.6 (1.7–34.3)
RBC density (%)	43.2 ± 10.4 (28.1–51.1)	39.0 ± 11.8 (19.6–53.3)	44.8 ± 6.9 (29.5–50.7)
Viscosity (cP)	2.5 ± 0.3 (2.0–2.8)	2.4 ± 0.4 (1.8–2.8)	2.5 ± 0.2 (2.1–2.7)
Shear rate (1/ms)	1.52 ± 0.60 (0.79–2.24)	2.68 ± 1.42 (1.64–6.08)	0.07 ± 0.03 (0.03–0.11)
Shear stress (dyn/cm^2^)	37.1 ± 11.7 (20.0–45.9)	63.6 ± 35.0 (37.4–144.7)	1.9 ± 0.9 (0.7–3.2)
**NE reactivity**			
% Change of Diameter (%)	−80.5 ± 73.0 * (−189.1– −36.2)	2.6 ± 14.5 (−21.0–24.0)	8.0 ± 11.0 (−8.4–25.6)

The combinations of vascular markers that were used to classify BM blood vessels during immunohistochemical (IHC), flow cytometric (FCM) and *in vivo* imaging (IVI) analysis in this study. Numbers are means ± SD, ranges are shown in parentheses. Asterisk indicates p < 0.05 (PBS vs. NE, paired t-test). These data obtained from four arteries, eight arterioles and eight sinusoids in five mice.

### Small molecules locally administered to mouse skull transit to calvarial BM

**Figure 2:**
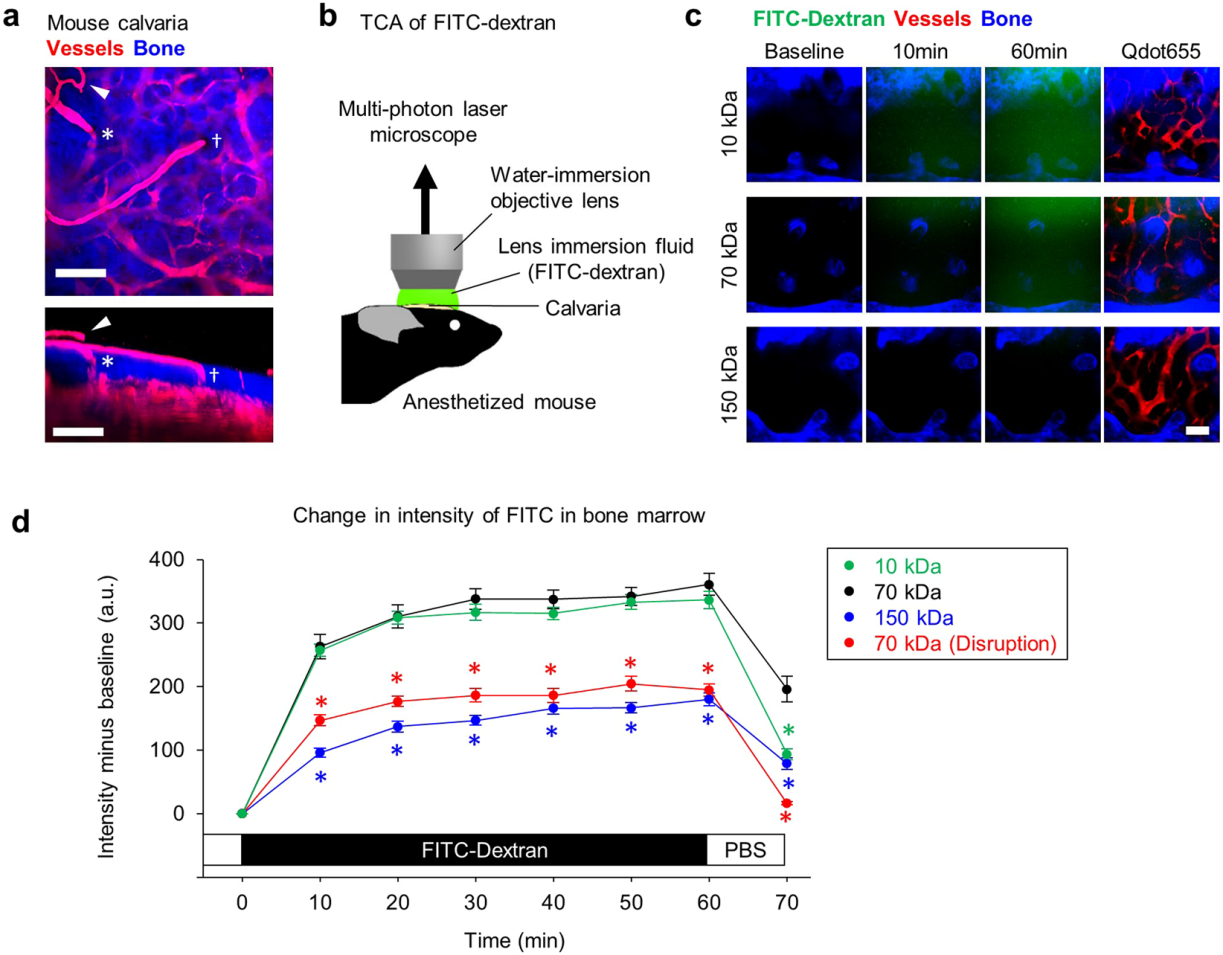
Intravital imaging of calvarial BM after local administration of fluorescein-conjugated dextran using the TCA technique. (**a**) A Z-stack multi-photon image from the outer surface of calvariato the BM cavity (upperpanel) and the corresponding rotatedimage (lower panel). Periosteal vasculature (arrowhead) and penetrating vessels on the outer surface of calvaria that dive into the BM cavity (at the asterisk and dagger). Scalebars = 100 μm. (**b**) Schematic representation of the TCA experimental setup. (**c**) Representative multi-photon imaging of mouse calvarial BM following TCA of FITC-conjugated 10 kDa (upper row), 70 kDa (middle row) and 150 kDa (lower row) dextran. To visualize blood vessels, Qdot655 was administered by i.v. injection at the end of the experiment. Scale bars = 100 μm. (**d**) Changes in FITC intensity following TCA of FITC-conjugated 10 kDa (green), 70 kDa (black) and 150 kDa (blue) dextran in calvarial BM. Red circles indicate FITC intensity following TCA of FITC-conjugated 70 kDa dextran after disruption by drying of the periosteal circulation. *p < 0.05 compared with 70 kDa. Values are means ± SEM. (n = 24, from 4 mice each).

We next focused on contractile function of AF633^+^ vessels as a potential mechanism regulating BM blood flow. To determine whether calvarial AF633^+^ vessels were contractile, we developed a method to locally administer vasoactive substances to calvarial BM. A fine reticulated vascular network is present on the skull outer surface, and this network penetrates calvarial BM, suggesting that transcalvarial vessels connect the outside with the inside of the BM (Fig. 2a). We dissolved various reagents in lens immersion fluid and then applied that solution directly to the skull prior to performing intravital imaging of blood vessels in the BM. (Fig. 2b). To confirm that reagents dissolved in lens immersion solution would penetrate small vessels and reach the calvarial BM (Fig. 2a,b), we added FITC-conjugated 70-kDa dextran to the fluid applied to the skull. Then, we monitored changes in fluorescence intensity in calvarial BM, as intravenously injected 70-kDa dextran reportedly leaks from the vasculature and diffuses into the BM cavity with time^18^. FITC intensity in the BM cavity increased immediately after dextran administration and remained high for 60 minutes (Fig. 2c,d). While FITC intensity increased after TCA of 10-kDa dextran, similarly to what we observed using 70-kDa dextran, FITC intensity was lower than that seen after administration of 70-kDa dextran 10 minute after FITC-dextran was washed out with PBS (Fig. 2c,d). In contrast, when we used 150-kDa dextran, FITC intensity 60 minutes later was lower than that seen with 70-kDa dextran at the same time point (Fig. 2c,d), suggesting that soluble molecules can be delivered into calvarial BM via lens immersion fluid and that delivery efficiency decreases in a molecular weight-dependent manner.

To investigate effects of charge on compound migration into BM during TCA, we compared the intensity of anionic versus cationic FITC-conjugated dextran in calvarial bone marrow. The intensity of both diethylaminoethyl (DEAE)-conjugated (cationic) and carboxymethyl (CM)-conjugated (anionic) 70 kDa-FITC-dextran was lower than that of unconjugated 70 kDa-FITC-dextran following 60 minutes of TCA (Fig. S2a). While the rate of change in fluorescence intensity in the proximal area was greater than that in the distal area at the onset of TCA and during wash-out of FITC-dextran, fluorescence intensity at both proximal and distal levels was comparable after 60 minutes of treatment with FITC-dextran (Fig. S2b). Moreover, Dimethyl sulfoxide (DMSO) and ethanol facilitated wash-out of 70 kDa-FITC-dextran (Fig. S2c).

To identify the delivery route of molecules from skull surface into the BM, we added FITC-70-kDa dextran after disrupting the skull microcirculation by 30 minutes of drying the skull surface. (Fig. S2d,e). Increases in FITC fluorescence seen after addition of FITC-70-kDa dextran on this disrupted surface were smaller than increases seen in intact groups from 10 to 60 minutes after FITC-dextran administration (Fig. 2d). Ten minutes after replacement of the lens immersion fluid containing FITC-dextran by PBS, the FITC signal in BM of mice that had undergone disruption of the circulation decreased to almost the same intensity value as baseline, while FITC intensity of intact mice remained higher than baseline (Fig. 2d). These data indicate that FITC-dextran diffusion from the skull surface to calvarial BM depends at least in part on transcalvarial blood flow via vessels penetrating the skull bone. Therefore, the calvarial BM microenvironment, including the vasculature, can be manipulated by TCA of small molecules.

### TCA of norepinephrine constricts arteries in calvarial bone marrow

To investigate whether microcirculation of calvarial BM could be manipulated by TCA of vasopressor substances, we measured changes in the diameter of AF633^+^ blood vessels after TCA of a thromboxane A2 receptor agonist (U46619), which has been used for experimental vasoconstriction in recent *ex vivo* studies^34,35^. TCA of 100 μM U46619 constricted A633^+^ blood vessels without markedly changing mean arterial pressure (MAP) or heart rate (HR) (Fig. S3a,b).

**Figure 3:**
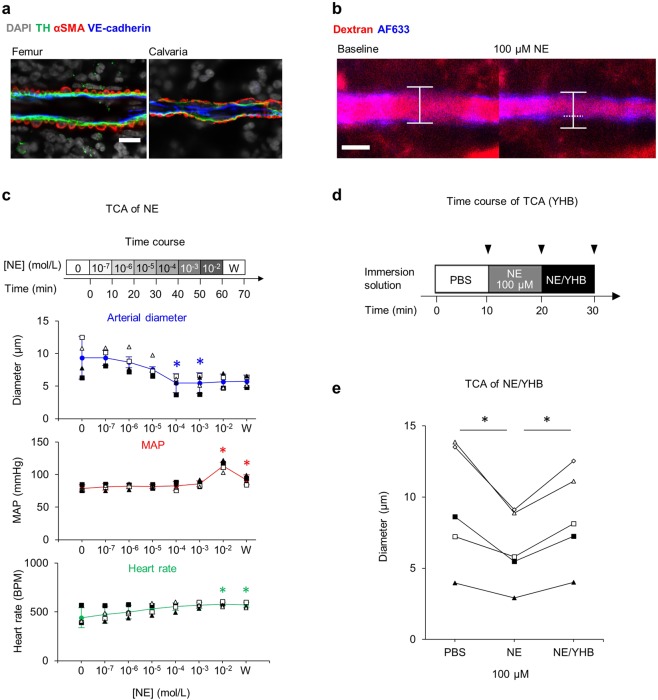
Changes in arterial diameter during TCA of NE. (**a**) TH expression in α-SMA-positive vessels in femoral and calvarial BM. Bar = 10 μm. (**b**) Representative arterial contraction 10 minutes after TCA of 100 μM NE as compared to the baseline state. Bar = 10 μm. (**c**) Focal and systemic changes during TCA of NE. Arterial diameter of calvarial BM, mean arterial pressure (MAP) and heartrate following treatment with 10^−7^ to 10^−2^ mol/L NE, as indicated. Values from independent experiments are each shown by open squares, closed squares, open triangles and closed triangles. Ws at right side of x-axis mean the values of 10 minutes after wash-out with PBS. (**d**) Time course of TCA with NE only and with NE plus Yohimbine (YHB). (**e**) Changes in diameter of AF633^+^ vessels during TCA of NE with and without YHB. Values from independent experiments are each shown by open squares, closed squares, open triangles, closed triangles and open diamonds. *p < 0.05 compared with baseline. Values are mean ± SD.

Because sympathetic nerves innervate the femoral BM^8,36,37^, we asked whether BM blood flow was locally regulated by adrenergic signaling in calvarial BM. We initially observed tyrosine hydroxylase (TH) signaling in the basement membrane of α-SMA^+^ vessels in calvarial BM (Fig. 3a), suggesting sympathetic innervation around calvarial arteries. We then treated calvarial BM with various concentrations of norepinephrine (NE) using TCA. As we increased concentration of NE in lens immersion fluid, we observed that arteries in calvarial BM contracted when that concentration reached 100 μM (Fig. 3b,c, Table 1, Supplementary Video), a point at which MAP and HR remained almost the same as baseline values (Fig. 3c). By contrast, intravenous injection of 10^−11^–10^−6^ mol of NE did not promote vasoconstriction in calvarial BM but significantly altered MAP and HR (Fig. S3c,d). These data indicate that the microenvironment of calvarial BM can be manipulated by TCA of drugs without an overt systemic effect.

To determine whether arteriolar constriction in calvarial BM was attributable to TCA of NE, we added yohimbine, an α2 adrenoreceptor antagonist, to NE in lens immersion fluid 10 minutes after TCA of NE only (Fig. 3d). Reduced arteriole diameter seen after treatment with NE alone returned to baseline levels following TCA of both yohimbine and NE (Fig. 3e), indicating that local blood flow in the BM is regulated by adrenergic signaling, which controls arterial diameter.

### Transendothelial migration of lymphocytes into decreases after TCA of NE

**Figure 4:**
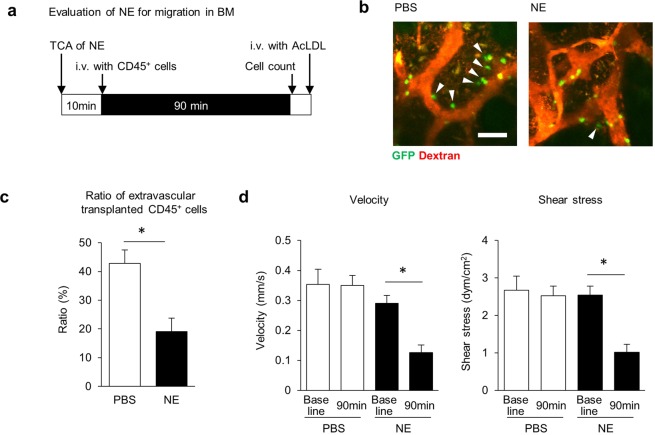
Evaluation of transendothelial migration of transplanted CD45^+^ BM cells after TCA of NE. (**a**) Schematic showing time course of TCA of NE and transplantation of GFP^+^ CD45^+^ BM mononuclear cells. (**b**) Migration of GFP^+^ CD45^+^ cells into calvarial BM 90 minutes after transplantation of GFP^+^ CD45^+^ cells following TCA of NE or PBS control. Arrowheads indicate extravasated GFP^+^ CD45^+^ cells. Bar = 50 μm. (**c**) Ratio of extravasated GFP^+^ to total GFP^+^ cells in region of interest (500 μm × 500 μm) 90 minute after transplantation of GFP^+^ CD45^+^ cells. (**d**) Red blood cell velocity and shear stress insinusoids in calvarial BM at baseline and at 90 minutes after transplantation of GFP^+^ CD45^+^ cells following TCA of NE. n = 9, *p < 0.05. Values are means ± SEM.

Lymphocyte migration and HSPC homing are reportedly important to maintain the immune response and hematopoiesis, respectively^38,39^. To assess a potential role for NE in blood cell migration into the BM, we first performed TCA of NE and 10 minutes later injected mice i.v. with CD45^+^ cells sorted from Ubc-GFP mice. We then counted extravasated GFP^+^ cells 90 minutes after injection (Fig. 4a). At that 90-minute time point, the number of GFP^+^ cells was comparable in NE and PBS control (Fig. S4a). However, the ratio of extravascular GFP^+^ CD45^+^ cells to total GFP^+^ cells in NE-treated mice BM was lower than that seen in PBS control mice (Fig. 4b,c). A recent report shows that shear stress facilitates transendothelial lymphocyte migration^40^; therefore, we compared sinusoidal shear stress in BM of NE-treated versus PBS-treated mice. For that analysis, we detected sinusoids by AcLDL incorporation into the vessel wall after counting of migrated GFP^+^ cells (Figs. 4a and S4b). Blood velocity and shear stress in sinusoids were decreased by NE treatment without marked changes in sinusoidal diameter or viscosity (Figs. 4d and S4c). These results suggest that sympathetic signals attenuate migration/homing efficiency of blood cells by down-regulating BM blood flow. These findings indicate overall that TCA can be used not only to promote vasoconstriction but to alter cell movement in calvarial BM.

## Discussion

The BM microenvironment is physiologically hypoxic^41–43^. Nonetheless, BM blood cells including HSPCs require a sufficient supply of oxygen and nutrients. In addition, wall shear stress in the BM vasculature due to local blood flow contributes to blood homeostasis by regulating transendothelial migration of HSPCs and differentiated cells between the circulation and the BM cavity^19^. Therefore, understanding homeostasis of the blood system requires knowledge of how the BM vascular network is organized and regulated. Intravital imaging of calvarial BM has helped define the spatiotemporal dynamics of BM vasculature and hematopoietic cells including HSPCs^17–19,26^. Three types of BM vasculature—arteries, arterioles, and sinusoids—contribute to blood homeostasis, although in different ways. For example, sinusoidal vessels occur at sites where blood cells migrate between the circulation and the BM cavity via transendothelial migration^2,19^. Sinusoidal endothelial cells also provide HSC niche factors^2^. Here, to advance BM imaging techniques, we first prepared a method to identify vessel types in calvarial BM by intravital staining without using a genetic reporter. Employing both AF633 and fluorophore-labelled anti-Sca-1 antibody and AcLDL, we simultaneously identified arteries, arterioles, and sinusoids in calvarial BM of live mice (Fig. 1). We next confirmed that a small molecule compound in lens immersion fluid could diffuse into the BM cavity via transcalvarial vessels (Fig. 2). Using this method, we manipulated contraction of BM vessels by application of norepinephrine without systemic effects in the circulation (Fig. 3). In this study, we used 100 μM NE for TCA. To increase concentration of NE in BM to 100 μM by i.v. injection, at least 2.5 × 10^−10^ mol of NE is required in case in which total blood volume of mouse is 2.5 mL. As Fig. S3d shows that i.v. injection of 2.5 × 10^-10^ mol NE increases MAP and heart rate without marked change of arterial diameter in BM. Thus TCA, but not i.v. administration, is suitable for local manipulation of calvarial BM environment. We also evaluated effects of NE in lens immersion fluid on transendothelial migration of blood cells using a fluorescent protein reporter mouse (Fig. 4). These approaches provide a means to identify and manipulate calvarial BM vessels and advance our understanding of spatiotemporal regulation of the BM vasculature and transplanted cells. BM sympathetic nerve and adrenergic signals reportedly alter HSPC dynamics either indirectly by regulating mesenchymal niche cells or directly by regulating HSPCs themselves^8,39,43^. Our findings further indicate that adrenergic signals from sympathetic nerves regulate local blood flow by inducing arterial vasoconstriction in BM. As the number of arteries decreases with aging, physiological hypoxia of the HSC niche is disrupted and remodeled to form a pathologically hypoxic environment seen in the aged BM^44^. Thus analysis of the BM vasculature following manipulation by TCA can reveal processes associated with BM aging. To expand application of the TCA method, it is now necessary to determine which types of compounds most efficiently diffuse into the BM cavity to alter BM cell dynamics without systemic effects. Classification of these compounds and additional analysis may expand application of the TCA method. Technically, in the *in vivo* hematopoietic cell migration assay (Figs. 4 and S4), we first observed the movement of transplanted GFP^+^  hematopoietic cells and then measured blood flow in BM sinusoid identified by staining vascular labels including AF488-AcLDL (a sinusoidal label) (Fig. 4a). Although GFP^+^ cells and AF488^+^ dots are clearly distinguishable in size (Fig. S4b), use of different fluorescent colors for hematopoietic cells and vasculatures enables simultaneous dissection of these components at the same time.

In conclusion, we have established an identification/manipulation method of BM vessels in calvarial BM of living mice and identified a vasoregulatory function of local adrenergic signaling of BM. Our findings illustrate the importance not only of local blood flow regulation but also of various other hematopoietic events such as HSPC egress and homing in BM. These studies provide understanding of how hematopoiesis is locally modulated by the BM microcirculation and pO_2_ regulation.

## Methods

### Animals

C57BL/6 J micewere purchased from CLEA Japan. Ubc-GFP reporter mice were purchased from The Jackson Laboratory. Mice were housed under a 12-h light–12-h dark cycle (8 a.m. to 8 p.m.) and fed ad libitum a standard CE-2 diet (CLEA Japan). All experimental procedures were approved by the animal experiment committee of National Center for Global Health and Medicine Research Institute and performed in accordance with guidelines of National Center for Global Health and Medicine.

### Reagents

Alexa Fluor 488 AcLDL (L23380), Alexa Fluor 633 (A30634) and Qdot 655 (Q21021MP) were purchased from Life Technologies. DL-Norepinephrine hydrochloride (A7256), FITC dextran 10 kDa (FD10S), 70 kDa (46945), 150 kDa (FD150S), FITC-DEAE-Dextran 70 kDa (54702), FITC-CM-Dextran 70 kDa (54701) and TRITC-Dextran 500 kDa (52194) were obtained from Sigma. Yohimbine hydrochloride (36805-31) was purchased from NacalaiTesque. U-46619 (sc-201242) was obtained from Santa Cruz Biotechnology.

### Antibodies

The following antibodies were used in this study: TER-119 (BioLegend), CD45 (30-F11; BD Biosciences), CD31 (MEC 13.3; BD Biosciences), VE-cadherin (eBioBV13; eBioscience), Sca-1(E13–161.7; BioLegend), Podoplanin (8.1.1; BioLegend), α-SMA (1A4; Sigma), tyrosine hydroxylase (2792; CellSignaling), NG2 (AB5320; Chemicon) and vWF (RB-281-A0, NeoMarkers). AF488-conjugated anti-mouse IgG2a (A-21131), AF488-conjugated anti-rabbit IgG (A-11008), AF555-conjugated anti-rat IgG (A-21434), AF555-conjugated anti-mouse IgG2a (A-21137), AF633-conjugated anti-rat IgG (A-21094) were obtained from Thermo Fisher Scientific.

### Isolation of endothelial cells

Isolation of endothelial cells from mouse BM was performed as described by Xu *et al*.^32^ with slight modification. Frontal bone was minced in 1 ml digestion solution (7 mg/ml Collagenase type I (Gibco), 1 mg/ml DNase I (Sigma) inPBS/2% FCS) using scissors and incubated at 37 °C for 20 minutes. Digested BM was suspended by gentle pipetting, followed by filtration through 70-mm nylon mesh. Cells were washed by centrifugation in PBS/2% FCS and then prepared for flow cytometry.

### Flow cytometry

BM cells (BMCs) were isolated by digestion of calvarial BM of mice injected intravenously with fluorophore-labelled antibody against podoplanin 10 min before sacrifice. BMCs were stained with antibodies against surface markers including TER-119, CD45, CD31, VE-cadherin and Sca-1 at 4 °C for 30 min. Stained cells were analyzed by SORP FACSAriaIII (BD Biosciences). Data were analyzed using FlowJo^TM^ software (Tree Star Inc).

### Immunohistochemistry

For histological identification of BM vessels *in vivo*, frozen BM sections were prepared and immunostained according to the Kawamoto method^31,39^. Briefly, frozen BM sections were fixed in 4% PFA/PBS for 5 min, washed three times with PBS and then stained with primary antibodies (diluted 1:500) for 16 hours. Specimens were washed three times with PBS and stained with DAPI and fluorophore-labelled secondary antibodies (1:1000) for 4 hours at room temperature. All antibodies were diluted in Protein Block (Dako). Immunofluorescence data were obtained and analyzed with a confocal laser scanning microscope (LSM880; Zeiss).

### Intravital microscopy

Male mice (10–12 wks old, nonfasting) were used for experiments in this study. Mice were anesthetized by intraperitoneal injection of urethane (800 mg kg^−1^) and α-chloralose (80 mg kg^−1^), tracheotomized, and intubated with a handmade Y-shaped tube for mechanical ventilation. The left femoral artery was cannulated for sampling arterial blood for blood gas analysis, and an arterial catheter connected to a pressure transducer (MP 150; BioPac Systems) was placed in the left femoral artery to continuously monitor mean arterial pressure. Rectal temperature was maintained at 37.0 ± 0.5 °C throughout the experiment with a heating pad. Animals were mechanically ventilated with a small-animal ventilator (MiniVent type 845; Harvard Apparatus) with 21% O_2_ balanced by N_2_ at a tidal volume of 8 μL g^−1^ with a respiratory rate of 120 breaths min^−1^.

The two-photon microscope consisted of an upright microscope (BX61WI; Olympus) attached to a mode-locked titanium-sapphire laser system (Chameleon Vision II; Coherent) capable of achieving a 140-fs pulse width and an 80-MHz repetition ratewith 17 W of pump power, and an 800-nm laser was utilized. Images (512 × 512 pixels) were acquired with an Olympus FV1000 scanning unit using Fluoview software (version FV10-ASW; Olympus) and a 25× objective (XLPLN25 × WMP; NA 1.05). The maximum average power exiting the objective lens was reduced using an acousto-opticaltunable filter by tuning a transmissivity parameter usingFluoview software. Emitted fluorescence was detected with anexternal photomultiplier tube (R3896; Hamamatsu Photonics) after passing through an infrared-blocking filter (685-nm cutoff) and an emission filter (420–520 nm). Immunofluorescence data were analyzed by Fluoview software Ver.4.2 (Olympus).

Vascular diameter, RBC velocity and RBC density were measured by multi-photon microscopy as described previously^19,35,45^. Briefly, to visualize the calvarial BM microvasculature and to measure the diameter of them, five milligrams of TRITC conjugated-dextran (Sigma) was injected into femoral vein of anesthetized mouse. RBC velocity and density were measured by multi-photon microscopy in line-scan mode. These measured parameters were used to calculate RBC flux, blood viscosity, shear rate and shear stress by calculation method as previously reported^19^. NE reactivity of blood vessels was demonstrated as rate of change in vessel diameter 10 minute after transcalvarial administration of 100 μM of norepinephrine.

### Isolation of GFP^+^ CD45^+^ BM mononuclear cells

Femurs and tibiae of Ubc-GFP mice were flushed with PBS + 2% FCS using a 21-gauge needle and a 10 mL syringe to collect the BM plug, which was then dispersed by reflux through the needle. The suspension was centrifuged 680 × g for 5 minutes at 4 °C. Cells were then lysed with lysis buffer (0.17M NH_4_Cl, 1 mM EDTA, 10 mM NaHCO_3_) at room temperature for 5 minutes, washed with 2 volumes PBS + 2%FCS, and centrifuged at 680 × g for 5 minutes. Cells were resuspended in PBS + 2%FCS and filtered through 40 μm nylon mesh. Cells were again centrifuged 680 × g for 5 minutes and treated with anti-CD16/32 antibody for Fc-receptor block (2 μL/mouse) for 5 minutes followed by addition of anti-CD45 magnetic beads (Miltenyi) at a 1/5 volume/volume ratio for 15 minutes. After removing the antibody with two PBS + 2%FCS washes, CD45-positive cells were isolated using Auto-MACS Pro (Miltenyi). Isolated cells were centrifuged once at 340 × g for 5 minutes. and injected into WT mice (3 × 10^6^ cells/mouse).

### Statistical analysis

All values presented here are expressed as means ± SEM, unless noted otherwise. Differences between means were evaluated for significance using ANOVA followed by Fisher’sexact test for multiple comparisons. Differences with a p value < 0.05 were considered statistically significant.

## Supplementary information

Supplementary Video.

Supplementary Information.

## Data Availability

The datasets generated during and/or analysed during the current study are available from the corresponding author on reasonable request.
