# Supplementary material for: Identification and local manipulation of bone marrow vasculature during intravital imaging
Source: Sci Rep. 2020 Apr 14;10:6422. doi: 10.1038/s41598-020-63533-3 (PMC7156750; doi:10.1038/s41598-020-63533-3)

Supplementary Information

Identification and local manipulation of bone marrow vasculature during intravital imaging

Takayuki Morikawa, Shinpei Tamaki, Shinya Fujita, Makoto Suematsu and Keiyo Takubo

Supplementary Figure Legends:

Supplementary Figure S1. **Classification of blood vessels in calvarial and femoral BM.** (a) In vivo staining of NG2$^+$ vessels in BM by AF633. Shown is immunohistochemical localization of vascular NG2$^+$ cells and ECs in a BM section obtained from an AF633-injected mouse. Scale bars=50 μm. (b) Representative Sca-1$^+$ vessel in a slice of calvarial BM based on immunohistochemical staining with vascular markers. Closed arrowheads, open arrowheads and arrows indicate NG2$^+$ AF633^{high}, NG2$^+$ AF633^{mid} and NG2$^+$ AF633^{low} vessels, respectively. Bar=50 μm. (c) Immunohistochemical staining for vWF in calvarial BM. Em and VE indicate endomucin and VE-cadherin, respectively. Bar=10 μm. (d) Representative flow cytometry gating strategies used to identify AECs and SECs in femoral BMMNCs. Femoral BMMNCs were obtained 60min after i.v. injection of AcLDL and anti-Sca-1 antibodies. Numbers in the graph indicate MFI of Sca-1 of AECs and SECs (mean ± SD, n=3, *p<0.05). (e) Representative Sca-1$^+$ vessel in a slice of calvarial BM based on immunohistochemical staining with vascular markers. Closed arrowheads, open arrowheads and arrows indicate α-SMA^{high} AF633^{high}, α-SMA^{mid} AF633^{mid} and α-SMA^{low} AF633^{low} vessels, respectively. Bar=50 μm. (f) Histogram and MFI of AF488-conjugated AcLDL incorporated into AECs and SECs in femoral BM (mean ± SD, n=3, *p<0.05). (g) Expression of Scf, Efnb2,

Vcam1 and Sele mRNAs in AECs and SECs sorted using AF488-AcLDL (h) Representative image of calvarial bone marrow (BM), frontal bone (FB), parietal bone (PB), sagittal suture (SS) and coronal suture (CS). The green box indicates approximate location and direction of Fig1E. Inside of solid line is calvarial BM imaged in this study. Bar=1 mm (i) In vivo AF633 staining in calvarial BM. Parts of blood vessels (arrowheads) and the surface of bone (arrows) were AF633-positive. Bar=50 μm. (j) Transitional zone of artery and arteriole is visualized as $AF633^{+}Sca\text{-}1^{+}$ vessels (arrowheads). Bar=20 μm. (k) Transitional zone of arteriole and sinusoid is shown as $Sca\text{-}1^{+}AcLDL^{+}$ vessels (cyan box). Bar=20 μm.

Supplementary Figure S2. Changes in FITC intensity following TCA of FITC-conjugated dextran. (a) Changes in intensity following TCA of FITC-DEAE- (cationic, open circles) and FITC-CM- (anionic, closed circles) conjugated 70 kDa dextran in calvarial BM. FITC-conjugated 70 kDa dextran served as control (gray circles). $*p < 0.05$ compared with control; †$p < 0.05$ compared with cationic dextran, Values are means ± SEM. (n = 24, from 4 mice each) (b) Changes in FITC intensity following TCA of FITC-conjugated 70 kDa dextran at proximal (0 to 500 μm from sagittal suture) and distal (500 to 1000 μm from sagittal suture) in calvarial BM. $*p < 0.05$ Values are means ± SEM.

(n = 24, from 4 mice each) (c) Changes in FITC intensity after wash-out of FITC-conjugated 70 kDa dextran with PBS, DMSO and ethanol. *$p < 0.05$ Values are means ± SEM. (n = 18 to 24, from 3 to 4 mice each) (d) Representative line-scan images of periosteal blood vessels baseline and after disruption by drying. (e) RBC velocity in four periosteal blood vessels at before (baseline) and after disruption of periosteal circulation. Thirty minutes after removing lens immersion fluid from the mouse skull, blood flow at the skull surface stopped. Values from independent experiments are each shown by open squares, closed squares, open triangles and closed triangles.

Supplementary Figure S3. Effects of TCA of U49919 and i.v. NE administration. (a) Representative arterial contraction 3 minutes after TCA of 100 μM U46619 relative to the baseline state. Bar=10 μm. (b) Focal and systemic changes during TCA of NE. Arterial diameter of calvarial BM, mean arterial pressure (MAP) and heart rate following treatment with 10^{-8} to 10^{-3} mol/L NE, as indicated. Values of independent experiments are each shown by open squares, closed squares, open triangles and closed triangles. Ws at right side of x-axis mean the values of 3 minutes after wash-out with PBS. (c) Representative arterial contraction 10 minutes after i.v. NE administration as compared to the baseline state. Bar=10 μm. (d) Effects of i.v. administration of NE on parameters

of calvarial BM and systemic circulation. Arterial diameter in calvarial BM, mean arterial pressure (MAP) and heartrate were measured after i.v. injection of 10^{-11} to 10^{-6} mol of NE. Values of independent experiments are each shown by open squares, closed squares and closed triangles. *$p < 0.05$ compared with baseline. Values are means ±SD.

Supplementary Figure S4. Evaluation of transendothelial migration of transplanted $CD45^{+}$ BM cells after TCA of NE. (a) Number $GFP^{+}CD45^{+}$cells migrated into calvarial BM in a region of interest 90 minute after transplantation of $GFP^{+}CD45^{+}$ cells after TCA of NE. *$p < 0.05$ compared with control. (b) Shown are particles of AF488-conjugated AcLDL incorporated into sinusoidal vessel walls (arrows) and transplanted $GFP^{+}CD45^{+}$ cells (arrowhead) before (left) and after (right) injection of AF488-AcLDL. Bar=50 µm. (c) Diameter, flux, red blood cell density, viscosity and shear rate in sinusoids in calvarial BM at baseline and 90 minute after transplantation of $GFP^{+}CD45^{+}$ cells after TCA of NE. n=9, *$p< 0.05$. Values are means ± SEM.

Supplementary Video. Movie shows constriction of artery in calvarial BM by TCA of NE. Arrow indicates contracting AF633 (blue)-positive BM blood vessel from a mouse injected i.v. with TRITC-conjugated-500 kDa dextran (red). Marked changes in vascular

diameter are not seen in the AF633-negative vessel (arrowhead).

Supplemental Fig. S1

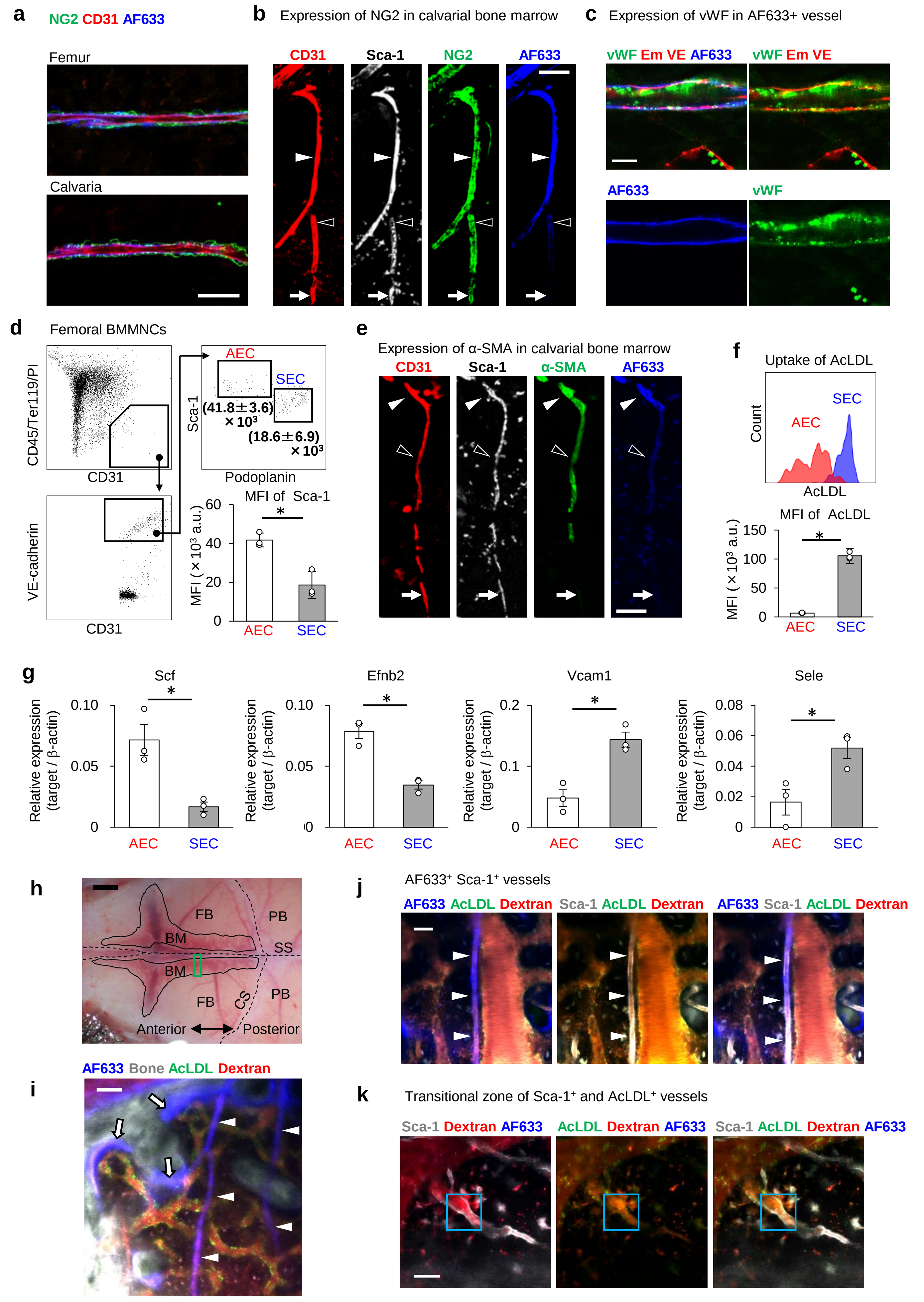

Supplemental Fig. S2

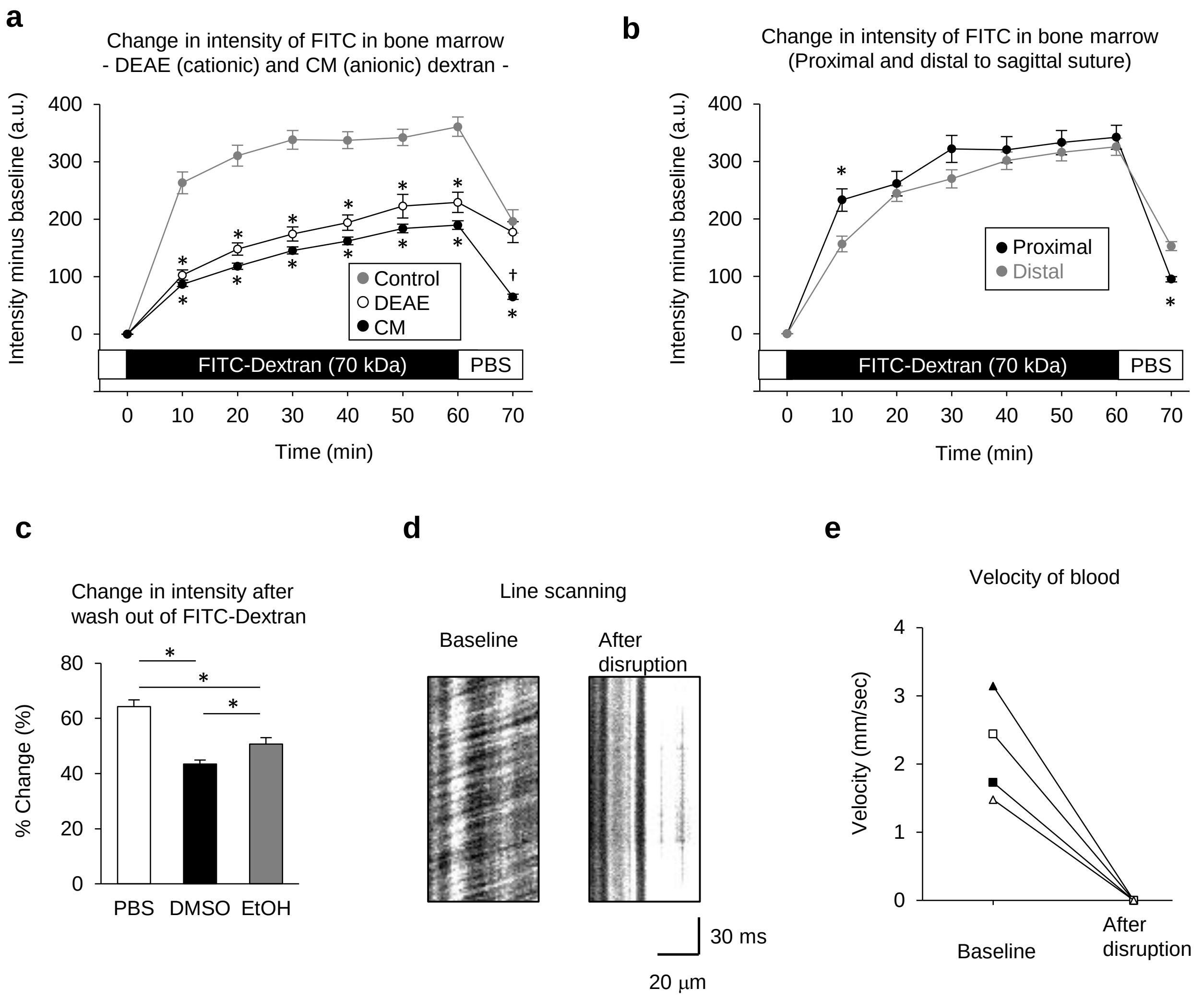

Supplemental Fig. S3

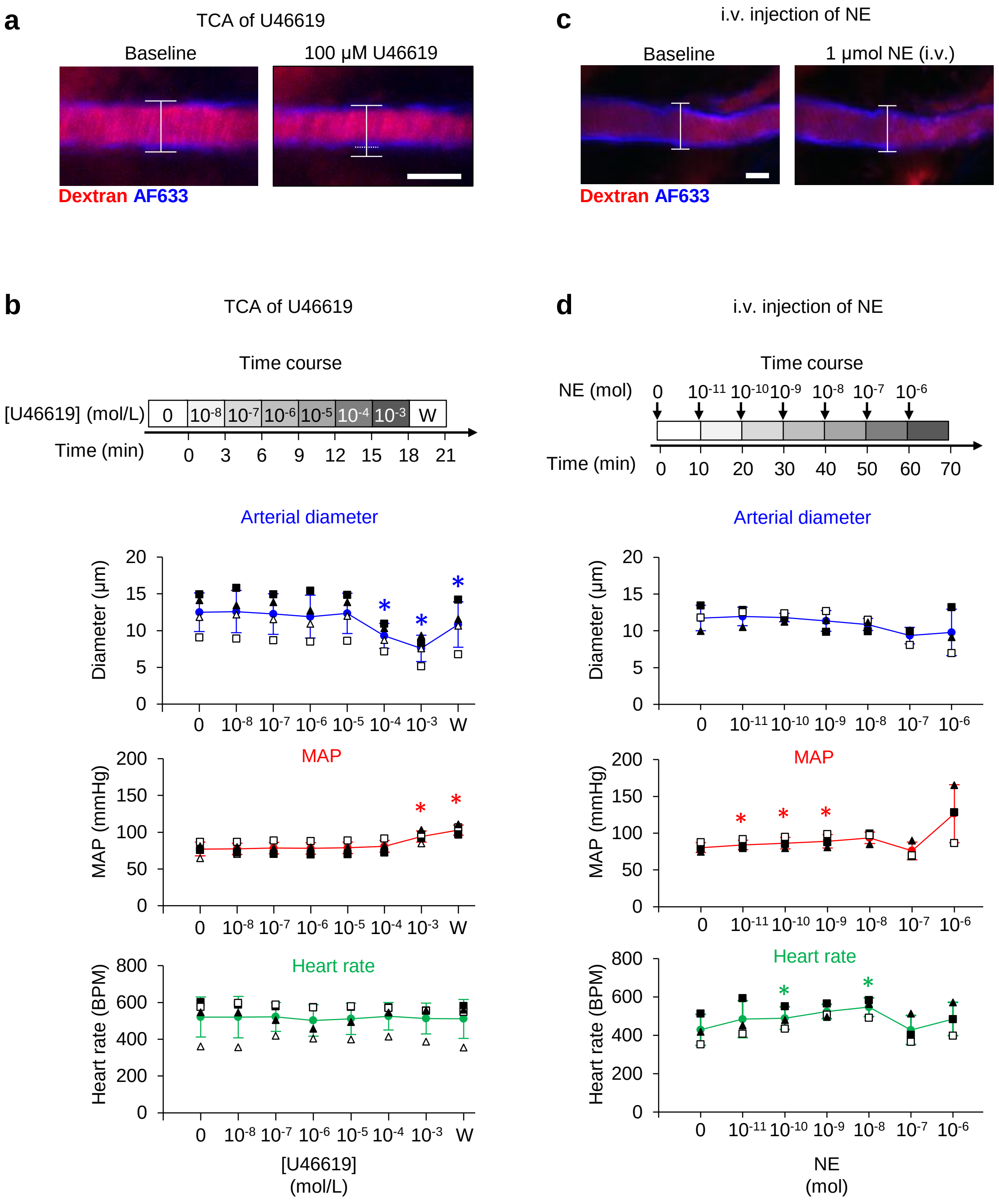

Supplemental Fig. S4

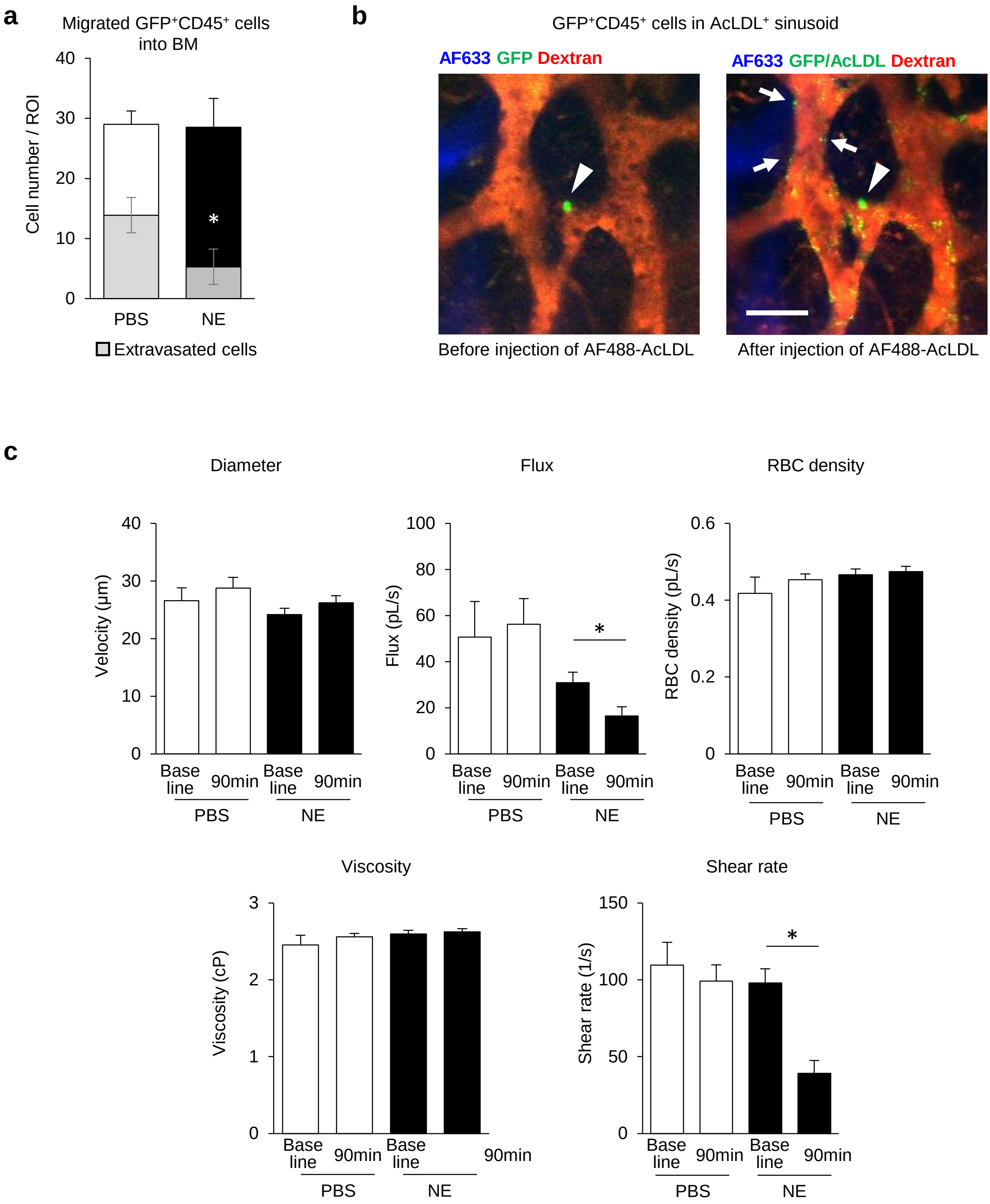

Supplement: Supplementary file 2 — Supplementary Information. [file 41598_2020_63533_MOESM2_ESM.pdf]
